# Phylogenetic Resolution and Quantifying the Phylogenetic Diversity and Dispersion of Communities

**DOI:** 10.1371/journal.pone.0004390

**Published:** 2009-02-05

**Authors:** Nathan G. Swenson

**Affiliations:** Center for Tropical Forest Science–Asia Program, Arnold Arboretum, Harvard University, Cambridge, Massachusetts, United States of America; Max Planck Institute for Evolutionary Anthropology, Germany

## Abstract

Conservation biologists and community ecologists have increasingly begun to quantify the phylogenetic diversity and phylogenetic dispersion in species assemblages. In some instances, the phylogenetic trees used for such analyses are fully bifurcating, but in many cases the phylogenies being used contain unresolved nodes (i.e. polytomies). The lack of phylogenetic resolution in such studies, while certainly not preferred, is likely to continue particularly for those analyzing diverse communities and datasets with hundreds to thousands of taxa. Thus it is imperative that we quantify potential biases and losses of statistical power in studies that use phylogenetic trees that are not completely resolved. The present study is designed to meet both of these goals by quantifying the phylogenetic diversity and dispersion of simulated communities using resolved and gradually ‘unresolved’ phylogenies. The results show that: (*i*) measures of community phylogenetic diversity and dispersion are generally more sensitive to loss of resolution basally in the phylogeny and less sensitive to loss of resolution terminally; and (*ii*) the loss of phylogenetic resolution generally causes false negative results rather than false positives.

## Introduction

The species list from a community may be used to provide two immediate indices depicting its biodiversity. The first is the number of species, or species richness, found in the community. The second is a measure of the taxonomic dispersion between co-existing species such as the genus to species ratio. While these two measures are still often reported and analyzed, conservationists and community ecologist have become increasingly interested in quantifying the phylogenetic diversity and phylogenetic dispersion of communities [1]–[12]. This is because both of these measures provide more detailed evolutionary information regarding the community composition than can be surmised from a list of Latin binomials. Phylogenetic branch lengths provide a continuous metric of relatedness while taxonomic levels provide an ordinal metric of relatedness [1], [9]. The enhanced level of detail provided by phylogenetic branch lengths has allowed for the quantification of phylogenetic diversity and dispersion. These more refined metrics of community biodiversity are now being applied for purposes ranging from delineating geographic regions as priorities for conservation [4], [7], [13] to understanding the ecological and evolutionary mechanisms that promote species diversity and co-existence [3], [9].

Despite this interest in quantifying the phylogenetic diversity and dispersion of communities, many methodological hurdles remain. First, ecologists and conservationists are often limited in their capacity to calculate such phylogenetic metrics due to a lack of phylogenetic hypotheses for the communities of interest. This barrier has resulted in ecologists and conservationists taking one of two pathways. The first pathway has been to not perform phylogenetic analyses and to quantify the species richness and taxonomic ratios of a community. The second pathway has been to construct a phylogenetic hypothesis using novel molecular data or to generate a phylogenetic supertree using previously published datasets.

Although the second pathway has the potential to garner a more quantitative and evolutionarily grounded metric of biodiversity, the researcher must still confront the possibilities of biased results due to uncertainty in the phylogenetic tree. The loss of statistical power in phylogenetic studies due tree uncertainty is not a new problem. Comparative biologists have faced and are facing similar issues in the development of their methods [14]–[17]. There are three potential issues that have been confronted in the comparative biology literature that must also be confronted in the phylogenetic diversity and dispersion literature. First, the branch lengths of the phylogenetic hypothesis are only estimates and may not represent the true degree of relatedness. Uncertainty in branch length estimates has been reported to reduce the statistical power of phylogenetically independent contrasts, but not to an enormous degree [15], [18]. The issue of branch lengths likely biases estimates of phylogenetic diversity and dispersion. For example, if the relative branch lengths are not consistent using multiple methods, then one method will likely provide more or less phylogenetic diversity and dispersion due to shifts in the relative timing of diversification within clades in the phylogeny. The second issue is the depiction of sister taxa in the phylogenetic hypothesis is assumed to be correct when this may not be the case. This loss of power due to this uncertainty is expected to be severe in comparative analyses as it breaks central assumptions used in methods such as independent contrasts that assume the topology is true [14], [16]. The loss of power due to this uncertainty is also likely severe for those quantifying phylogenetic diversity and dispersion. The third potential issue is that few to many of the nodes within the phylogenetic hypothesis may be represented as ‘soft’ polytomies and the relatedness of descendent taxa is therefore unknown. The comparative biology literature on this topic is deeper, focusing on developing methods to deal with soft polytomies by representing the branch lengths between the lineages derived from a polytomous node as a zero [14], by collapsing the polytomous node to represent a single comparison or contrast [15], [17], [19] or by calculating *n*−1 contrasts [20].

**Figure 1:**
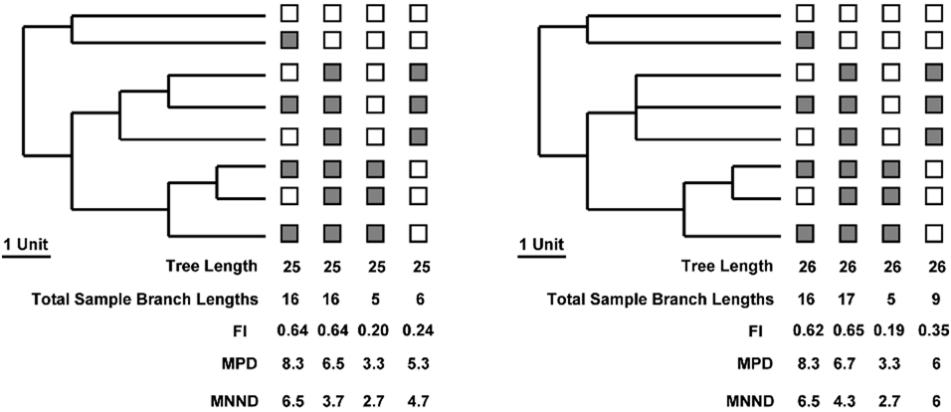
A graphical example of potential ways in which polytomies in the phylogenetic tree may influence different metrics of community phylogenetic diversity. The boxes indicate whether or not that species is found in the community. If the box is shaded grey, then the species is present in the community. If the box is not shaded, then the species is absent from the community. The left panel is a fully resolved phylogenetic tree and the three different measures of phylogenetic diversity (MPD, MNND, and FI) using four example assemblages. The MPD is the mean pair-wise phylogenetic distance between all taxa in the assemblage. The MNND is the mean nearest phylogenetic neighbor distance for all taxa in the assemblage. The FI (Faith's Index) quantifies the shared branch lengths between species in an assemblage as a proportion of the total branch lengths in the species pool phylogeny. The right panel shows the same phylogeny with one node now a polytomy and the same measures of phylogenetic diversity using this less resolved phylogeny with an increased total branch length. In all cases the FI measured is influenced as it represents a proportion of the total branch length. The MPD and MNND metrics are not influenced if the polytomy does not include species in the assemblage, but if it does include species in the assemblage these metrics may artificially increase or decrease.

In this article, I focus on the issue of soft polytomies and their potential to bias metrics of phylogenetic diversity and dispersion. I have chosen this focus because ecologists are increasingly generating phylogenetic supertrees in their work that contain multiple soft polytomies [6], [11]–[12], [21], [22]. Although this approach has allowed for phylogenetic analyses of species rich assemblages that have little pre-existing molecular or phylogenetic information, it remains unclear how much information and statistical power is lost in these studies compared to those using a fully bifurcating phylogenetic tree. On the one hand, the lack of resolution in the phylogeny may result in the researcher underestimating the phylogenetic diversity in communities due to the loss of terminal resolution and an increase in the total phylogenetic tree length. This scenario would be particularly plausible if the unresolved nodes do not subtend the taxa in the assemblage (Fig. 1). On the other hand, we may expect a researcher to overestimate the phylogenetic diversity in communities due to the increase in the total phylogenetic tree length. This scenario would be more likely if the unresolved nodes subtend many terminal taxa in the assemblage of interest (Fig. 1). Thus, there are opposing predictions regarding the relationship between the degree of phylogenetic resolution and metrics of phylogenetic diversity. Further, the influence of phylogenetic resolution on metrics of phylogenetic dispersion has not been quantified and *a priori* predictions are less clear because phylogenetic dispersion is quantified using randomizations that may mitigate the loss of statistical power and the potential to over- or under-estimate phylogenetic diversity and dispersion.

Given the increasing interest in quantifying the phylogenetic diversity and dispersion in communities and the increasing use of phylogenetic supertrees to conduct such measurements, it is critical that we quantify the potential biases and the potential loss of statistical power introduced by this approach. The present study is designed to provide such insights. Specifically, it starts by quantifying the phylogenetic diversity and dispersion in communities using a fully resolved phylogeny. Then it compares those values to those for the same communities using a phylogeny that is gradually ‘unresolved’ using two different methods. The results are used to address the following questions: (*i*) how correlated are the phylogenetic diversity and dispersion values generated using a fully resolved phylogeny to those generated using a phylogeny containing polytomies?; (*ii*) is the phylogenetic diversity and dispersion generally over- or under-estimated when using a phylogeny containing polytomies?; (*iii*) are metrics of phylogenetic diversity and dispersion less powerful as the number of polytomies in the phylogeny increases?; (*iv*) is the power to detect the known phylogenetic diversity and dispersion influenced more by basal or terminal polytomies?; (*v*) are metrics of phylogenetic diversity and dispersion less powerful as the number of species in the community relative to the number of species in the phylogeny increases?; and (*vi*) are different metrics of phylogenetic diversity and dispersion equally sensitive to the above conditions.

## Methods

### Phylogeny Generation

The present study was designed to quantify the degree to which polytomies in phylogenetic trees influence measures of phylogenetic diversity and dispersion in communities. To achieve this, I first randomly generated fully resolved ultrametric phylogenies using a uniform Yule-Harding branching process using the software PDA - Phylogenetic Diversity Algorithm Version 0.5 [23](http://www.cibiv.at/software/pda/) with the number of terminal taxa being 20, 40, 80, 160 or 320. Five random phylogenies were generated for each number of terminal nodes thereby providing the 25 fully bifurcating phylogenetic trees used in this study. These trees represented the species pool from which community assemblages were drawn. A Yule-Harding branching process with constant birth rates through time was used as a first step towards uncovering biases in the methods analyzed in the present study and it serves as a satisfactory model [24]. It is noted that a Yule-Harding processes may provide phylogenies that may be unrealistically balanced. Further, this method did not allow for analyzing the relative influence of decreases or increases in lineage diversification through time. *A priori* it would be expected that a decrease in diversification through time would reduce statistical bias and increase statistical power and an acceleration in lineage diversification through time would likely increase bias and reduce statistical power.

### Community Assemblage Generation

The community assemblages used in this study had species diversities that were 10, 15, 20, 25, or 30 percent of the number of terminal taxa in the phylogenetic trees representing the species pools. The assemblages were generated using three different methods. The first method was designed to generate the assemblage with the maximum possible phylogenetic diversity given a species richness. These assemblages were generated using the “Greedy Algorithm” [23], [25] implemented using the software PDA - Phylogenetic Diversity Algorithm Version 0.5 [23]. The Greedy Algorithm uses a phylogenetic tree and an assemblage species richness to output the species assemblage with the maximum total phylogenetic diversity. The second method was designed to generate the assemblage with the minimum possible phylogenetic diversity given an assemblage species richness. These assemblages were generated using a dynamic programming algorithm implemented in the software PDA - Phylogenetic Diversity Algorithm Version 0.5 [23]. The last method randomly drew species from the species pool. Specifically, thirty random assemblages were drawn from a species pool for a given assemblage species richness.

### Measurement of Phylogenetic Diversity and Dispersion

The phylogenetic diversity of the assemblages was measured using three methods commonly used by ecologists. The first measure was Faith's Index [1], which reports the shared branch lengths between species in an assemblage as a proportion of the total branch lengths in the species pool phylogeny. Faith's Index does not include the root connecting all taxa within the community to an outgroup. An alternative metric, Evolutionary History, commonly used by conservation biologists [e.g. 5] that does include the root in the calculation of phylogenetic diversity was not considered in the present study. As both Faith's Index and Evolutionary History have been called Phylogenetic Diversity or PD in the past, I have decided to abbreviate Faith's Index as FI in order to avoid confusion with other known metrics of phylogenetic diversity. The second method reports the mean pair-wise phylogenetic distance (MPD)[6] between species in the assemblage. The third method used was the mean nearest phylogenetic neighbor distance (MNND)[6] for the species in the assemblage.

The phylogenetic diversity of assemblages is generally correlated to species richness. At the same time community ecologists are also interested in whether the phylogenetic diversity in an assemblage is greater or less than that expected given the assemblage species richness. This is termed here as the phylogenetic dispersion of an assemblage. Two commonly used metrics were used in this study to quantify the phylogenetic dispersion of assemblages. Specifically, the Net Relatedness Index (NRI) and Nearest Taxon Index (NTI) of Webb and colleagues [6], [9] were calculated as follows:
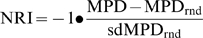
and
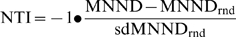
Where, 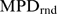 and 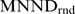 are the means of the MPD and MNND values from 999 randomly generated assemblages and the 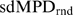 and 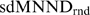 are the standard deviations of the 999 MPDs and MNNDs from those assemblages. Thus negative NRI and NTI values indicate a high level of phylogenetic overdispersion. In other words, negative NRI and NTI indicate higher than expected phylogenetic diversity in the assemblage given the species richness of that assemblage. The random assemblages generated in the null models were generated by drawing the same number of species from the pool as the number of species in the observed community and observed community occupancy rates were fixed, also known as an Independent Swap [26]. All calculations of NRI and NTI were made using the software Phylocom [27].

### Phylogenetic Resolution

Two methods were used to introduce soft polytomies into the original fully bifurcating phylogenetic tress. The first method used in this study was designed to randomly ‘unresolve’ internal nodes in the phylogeny. There were four different degrees to which the phylogeny was unresolved. Specifically, I randomly collapsed 15, 20, 25, and 30 percent of the internal nodes. The branch lengths for the edges subtended by the collapsed node were set to equal the length between the collapsed node and the next most terminal node in each lineage. This approach provided four phylogenies containing different numbers of polytomies for each original resolved phylogeny. This method was used to mirror a study where some basal nodes are unresolved, while at the same time some terminal clades have some nodes resolved.

The second method ‘unresolved’ the most terminal nodes on the phylogeny. Specifically, I collapsed 15, 20, 25, and 30 percent of the internal nodes in the phylogeny that were the most terminal on the phylogeny. The branch lengths for the edges subtended by the collapsed node were set to equal the length between the collapsed node and the tip of the tree. This method was used to simulate a scenario where species- or genus-level relationships are unknown, but the most basal nodes are bifurcating. The lack of resolution in more terminal internal nodes is common in studies using phylogenetic supertrees, but these studies also tend to have polytomous nodes basally as well [6], [11]–[12].

### Statistical Analyses

**Figure 2:**
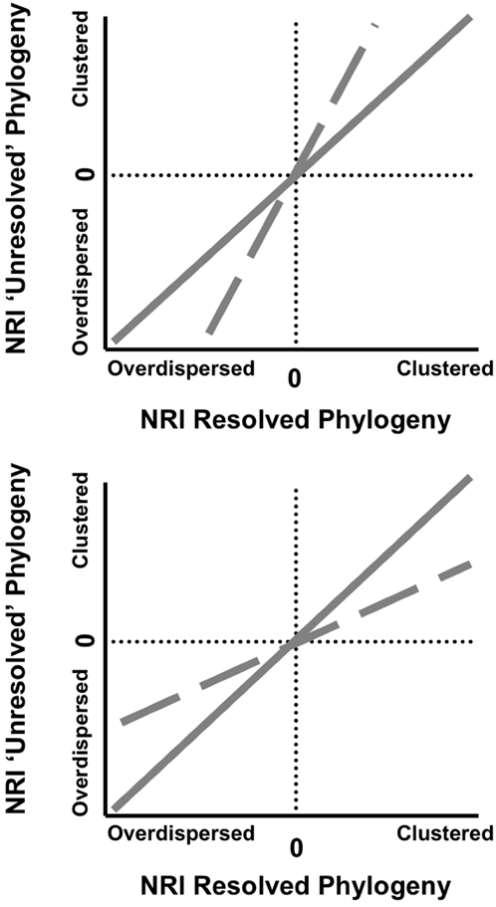
A graphical example how potential directional biases in phylogenetic dispersion produced by using phylogenetic trees containing polytomies were quantified in this study. The value for a phylogenetic dispersion metric, in this example NRI, generated for a community using a phylogeny containing polytomies is regressed through the origin onto the NRI value generated from the same community using a fully resolved phylogeny (dashed lines). As in the above example, the expected relationship is a 1∶1 line through the origin (Solid Line). When the slope is greater than one (dashed line in the top panel) shows a bias towards higher phylogenetic overdispersion and phylogenetic clustering. In other words, a bias towards non-random phylogenetic structuring (False Positives; Type I Error). When the slope is less than one (dashed line in the bottom panel) this shows a bias towards lower phylogenetic overdispersion and phylogenetic clustering. In other words, a bias towards random phylogenetic structuring (False Negatives; Type II Error).

The first goal of this study was to determine the degree to which the phylogenetic diversity and dispersion in a community measured using a fully bifurcating phylogeny is correlated with the phylogenetic diversity and dispersion of the same community using a phylogeny with multiple polytomies. The second was to determine whether phylogenetic diversity and dispersion tended to be over- or under-estimated, false positives and false negatives respectively, when a less resolved phylogeny was used. In order to answer both of these questions, I regressed the phylogenetic diversity and dispersion metrics from the phylogenies with polytomies onto the phylogenetic diversity and dispersion metrics from the fully resolved phylogeny. The expectation was a perfect correlation with a regression slope of unity. Thus all regression lines were forced through the origin and the coefficient of determination and the slope of the regression line were recorded. The coefficient of determination was used to answer the first question as to how tightly the results from the less resolved and fully resolved phylogenies were correlated. The regression slope was used to determine whether the results from the less resolved phylogeny tended to produce over- or under-estimates of the results from the fully resolved phylogeny. In Figure 2 I have provided a graphical example of this procedure.

## Results

### Phylogenetic Diversity

Three metrics of community phylogenetic diversity were used in this study. Specifically, I used: (*i*) the mean pair-wise phylogenetic distance between all taxa in an assemblage (MDP); (*ii*) the mean nearest phylogenetic neighbor distance for the taxa in an assemblage (MNND); and (*iii*) the proportion of the total branch lengths in the phylogeny represented in the assemblage excluding the root (Faith's Index: FI). The sensitivity of each of these three metrics to the resolution of the phylogenetic tree was quantified by ‘unresolving’ the phylogeny to varying degrees. There were a few general results from these analyses.

First, the correlation between the phylogenetic diversity in assemblages measured using a bifurcating phylogeny and a phylogeny with polytomies was generally strong (*r* ^2^>0.95) for all of the metrics analyzed (Table S1, S2, S3, S4, S5, and S6). The correlation was slightly weaker for all three metrics of phylogenetic diversity when the phylogeny was randomly ‘unresolved’ as compared to when only the most terminal nodes were ‘unresolved’ (Table S1, S2, S3, S4, S5, and S6). Second, as the phylogenetic tree contained an increasing number of unresolved nodes each observed phylogenetic diversity measure was highly correlated with the ‘known’ phylogenetic diversity, but the phylogenetic diversity tended to become slightly underestimated as the phylogeny became more unresolved (Table S1, S2, S3, S4, S5, and S6). Again, both of these effects were evident when the phylogeny was randomly and terminally ‘unresolved’. Third, as the number of terminal taxa in the phylogeny increased each metric tended to be more sensitive to the degree of phylogenetic resolution. For example, the phylogenetic diversity was increasingly underestimated in larger phylogenetic trees than smaller phylogenetic trees. Fourth, the above results were generally consistent across all non- randomly and randomly generated assemblages.

### Phylogenetic Dispersion

**Figure 3:**
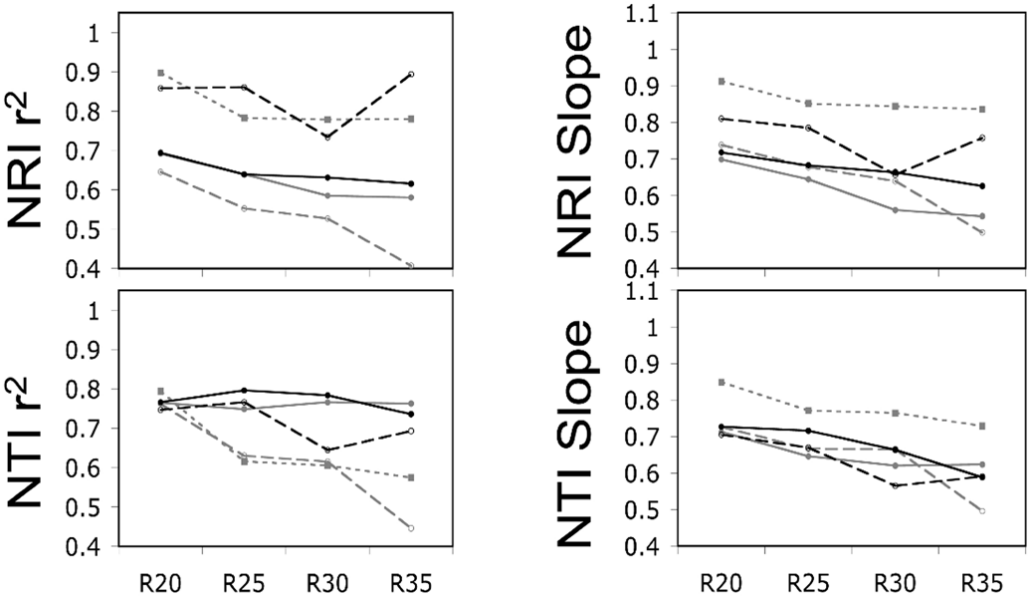
A figure showing the power to predict NRI and NTI of an assemblage with the maximal possible phylogenetic diversity estimated using the Greedy Algorithm. The slopes and r^2^ values from regressing the NRI and NTI values derived using a randomly ‘unresolved’ phylogeny onto the NRI and NTI values derived using a fully resolved phylogeny. The size of the phylogeny is represented by color and dashing of the lines. Specifically, the number of terminal taxa was 20 (finely dashed grey line), 40 (thickly dashed grey line), 80 (solid grey line), 160 (dashed black line), and 320 (solid black line). The percentage of nodes that were ‘unresolved’ is indicated by R_x_ on the x-axis. Slopes less than one show a bias towards under-predicting the phylogenetic diversity in an assemblage and vice versa for slopes greater than one (see Figure 1).

**Figure 4:**
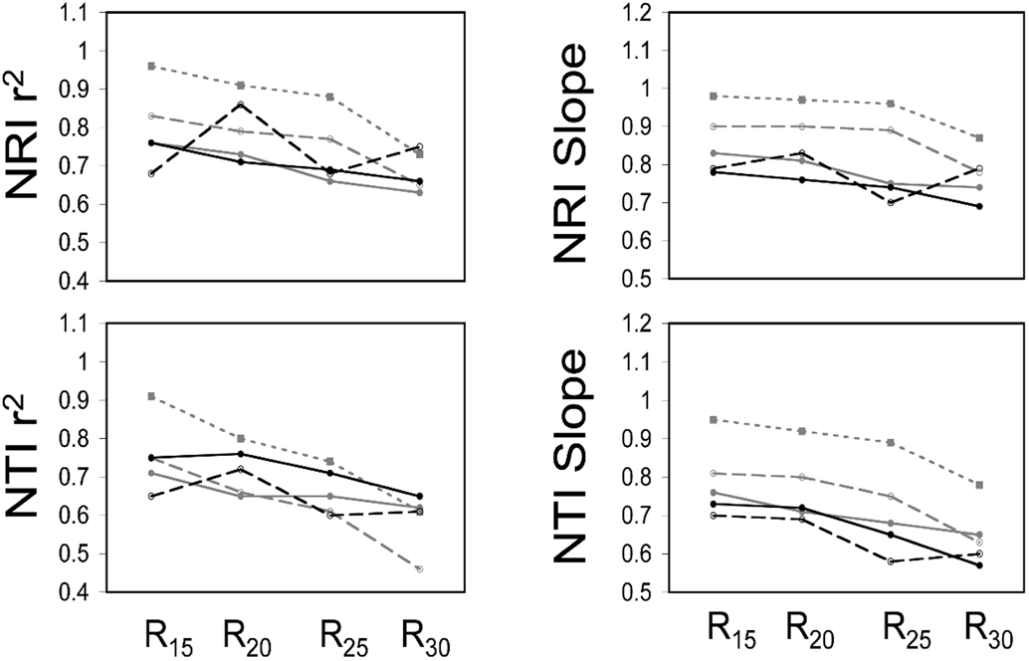
A figure showing the power to predict NRI and NTI of an assemblage with the minimal possible phylogenetic diversity estimated using a dynamic programming algorithm implemented in PDA. The slopes and r^2^ values from regressing the NRI and NTI values derived using a randomly ‘unresolved’ phylogeny onto the NRI and NTI values derived using a fully resolved phylogeny. The size of the phylogeny is represented by color and dashing of the lines. Specifically, the number of terminal taxa was 20 (finely dashed grey line), 40 (thickly dashed grey line), 80 (solid grey line), 160 (dashed black line), and 320 (solid black line). The percentage of nodes that were ‘unresolved’ is indicated by an R on the x-axis. Slopes less than one show a bias towards under-predicting the phylogenetic diversity in an assemblage and vice versa for slopes greater than one (see Figure 1).

**Figure 5:**
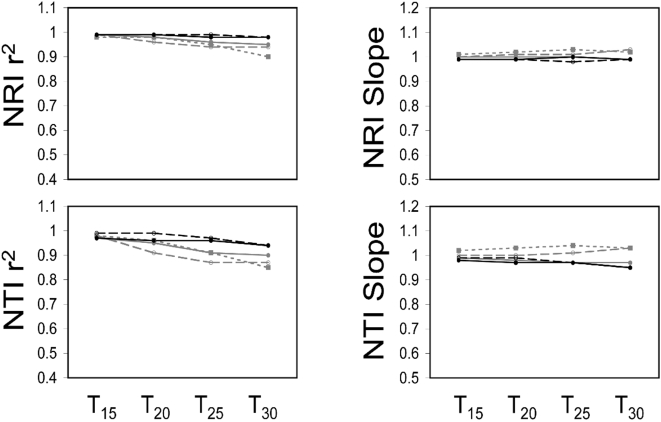
A figure showing the power to predict NRI and NTI of randomly generated assemblages. The slopes and r^2^ values from regressing the NRI and NTI values derived using a randomly ‘unresolved’ phylogeny onto the NRI and NTI values derived using a fully resolved phylogeny. The size of the phylogeny is represented by color and dashing of the lines. Specifically, the number of terminal taxa was 20 (finely dashed grey line), 40 (thickly dashed grey line), 80 (solid grey line), 160 (dashed black line), and 320 (solid black line). The percentage of nodes that were ‘unresolved’ is indicated by R_x_ on the x-axis. Slopes less than one show a bias towards under-predicting the phylogenetic diversity in an assemblage and vice versa for slopes greater than one (see Figure 1).

The present study tested the sensitivity of two commonly used metrics of phylogenetic dispersion, the Net Relatedness Index (NRI) and the Nearest Taxon Index (NTI), to varying degrees of phylogenetic resolution. When randomly ‘unresolving’ nodes on the phylogeny, the correlation between the NRI from the fully bifurcating tree and the NRI derived from phylogenies with polytomies generally became weaker as the phylogenetic resolution decreased (Fig. 3, 4, and 5). A similar result was found for the NTI, but the correlations were generally slightly weaker (Fig. 3, 4, and 5). The slope of the regression equations for NRI and NTI were generally always lower than one and decreased as the phylogenetic resolution decreased. Further, larger phylogenies tended to have shallower slopes than smaller phylogenies (Fig. 3, 4, and 5). In sum, the ability to predict the ‘known’ NRI and NTI decreased and the NRI and NTI values were generally closer to zero as the phylogenetic resolution decreased and phylogeny size increased (Fig. 2, 3, 4, and 5).

When ‘unresolving’ only the most terminal nodes on the phylogeny, the NRI and NTI in the assemblages were generally highly correlated (*r* ^2^>0.8) with the NRI and NTI values from the fully bifurcating phylogeny (Fig. S1, S2, and S3). There was a slight decrease in predictive power as the phylogenies were increasingly ‘unresolved’ and this was the most severe in the smaller phylogenies. The slopes of the regressions of the known NRI and NTI values against the NRI and NTI values from the ‘unresolved’ phylogenies were generally very close to one with a few greater than one (Fig. S1, S2, and S3). Thus, unresolving only the most terminal nodes in the phylogeny had much less influence on the power to predict the real NRI and NTI whereas randomly unresolving nodes on the phylogeny had a much greater negative impact. Further, the above results were consistent when the communities were non-randomly (Fig. 3 and 4) or randomly assembled (Fig. 5). Lastly, I investigated whether the number of taxa in an assemblage influenced the power to predict the known NRI and NTI. I found a weak trend showing that the power to predict the known NRI increased and the power to predict the known NTI decreased as the number of taxa in the assemblage increased (Fig. S4). This result was particularly noticeable in the largest phylogeny and again randomly unresolving the phylogeny had a much greater negative impact on predictive power than unresolving the most terminal nodes (Fig. S4).

## Discussion

In recent decades ecologists, evolutionists and conservationists have become increasingly interested in quantifying the phylogenetic diversity and phylogenetic dispersion of communities [1]–[3], [6], [9], [11]–[12], [21]–[22]. Despite this interest, quantifying the phylogenetic diversity and dispersion of communities often necessitates utilizing phylogenetic supertrees that contain multiple unresolved nodes. In this study I asked how does the use of a phylogeny with multiple polytomies bias commonly used metrics of phylogenetic diversity and dispersion.

The first part of this study focused on the phylogenetic diversity of randomly generated communities using three different metrics. Interestingly, the phylogenetic diversities recorded using phylogenies with polytomies were generally highly correlated with the phylogenetic diversity found for the same communities using a fully bifurcating phylogeny. Despite this strong correlation the phylogenetic diversity was increasingly underestimated as the phylogenies became less resolved and when larger phylogenetic trees were used. This was especially true when randomly unresolving nodes in the phylogeny. This is an intuitive result as this method unresolved more basal nodes in the phylogeny and therefore a larger number of terminal taxa were influenced. Lastly, Faith's Index (FI) tended to underestimate the known phylogenetic diversity to the greatest degree as the number of polytomies and phylogeny size increased followed by the MPD and MNND metrics. This result is most likely due to FI being represented as a proportion. For example, as the total phylogenetic tree length increased or decreased the FI will be altered even if the taxa subtended by the unresolved nodes are not in the community (Fig. 1). Conversely, the MPD and MNND should not be as influenced by unresolved nodes that subtend taxa not found in the community, but as the number of unresolved nodes increases, the probability that the MPD and MNND will be influenced increases as shown in the results (Table S1, S2, S3, S4, S5, and S6). Further as the number of nodes utilized in the calculation of MPD is generally higher than when calculating the MNND, MPD is expected to be more sensitive to the phylogenetic resolution. Indeed the results of this study supported these predictions (Table S1, S2, S3, S4, S5, and S6). It is important to note again that future sensitivity analyses should be performed on alternative metrics of phylogenetic diversity, such as Evolutionary History, that were not studied presently as the present results may not apply to those metrics. Future research should also aim to develop a set of methodologies that allow for closer estimates of the ‘true’ phylogenetic diversity of a community when the research must use a phylogenetic tree containing polytomies and the ‘true’ phylogeny is unknown. A potential way to accomplish this could be to randomly resolve the polytomies using each possible bifurcating topology, if feasible, and calculating the phylogenetic diversity metric using each of the potential topologies. This would generate a distribution of possible phylogenetic diversities from which a mean and 95% confidence intervals could be determined.

The second section of this study was designed to quantify the sensitivity of two commonly used phylogenetic dispersion metrics (NRI and NTI) to phylogenetic resolution. The NRI and NTI are calculated using the MPD and MNND respectively of the communities, but are standardized by the mean and variance of the MPD's and MNND's of the null assemblages. Thus, it has been unclear whether the NRI and NTI should be equally or less sensitive to the resolution of the phylogeny as compared to MPD and MNND. The results from this study show that both the correlation of the NRI and NTI measured using a fully resolved phylogeny and the NRI and NTI measured using a ‘unresolved’ phylogeny generally decreases as the phylogeny becomes less resolved (Fig. 3, 4, 5, S1, S2, and S3). The loss of predictive power is far greater when the phylogeny is randomly unresolved (Fig. 3, 4, and 5) compared to when the most terminal nodes were unresolved (Fig. S1, S2, and S3). Further, in most cases, the NRI and NTI quantified using less than fully resolved phylogenies were generally skewed more closely towards zero. This is shown by regressing through the origin the NRI and NTI data from the randomly unresolved phylogenetic analyses onto the NRI and NTI data from the fully resolved phylogenetic analyses where the regression slope is less than unity (Fig. 2, 3, 4, and 5). This trend shows that when using phylogenetic trees that are not completely resolved, a researcher is biased towards finding NRI and NTI values that are closer to the null expectation and there is a reduced power to detect non-random community phylogenetic structure. Further, this bias toward not finding non-random results (i.e. false negatives) was generally highest when using larger phylogenetic trees (Fig. 3, 4, 5, S1, S2, and S3). Converse to this pattern, when only the most terminal nodes of the phylogeny were unresolved the slopes from the NRI and NTI regression analyses were near one with small deviations above and below one. In particular, analyses using smaller phylogenies tended to have less power and were biased toward over-predicting non-random community phylogenetic structure (Fig. 3,4, and 5). The above results show that when large phylogenies are used containing polytomous nodes basally in the tree the researcher may expect to have substantially reduced statistical power to detect non-random phylogenetic community structure. While this loss of power is of concern, the results also suggest that if the most basal nodes are bifurcating and terminal nodes are unresolved the loss of power is greatly minimized. Thus for those constructing supertrees in the future for phylogenetic community analyses, the priority should be to attempt to resolve basal nodes prior to piecing together terminal topologies (i.e. con-generic relationships). For those that have used large phylogenetic supertrees with multiple soft polytomies in the past [6], [11]–[12], [21]–[22], it is likely that the results in such studies were biased towards finding random phylogenetic structure in the communities analyzed. This would be particularly true for studies that had species pool phylogenies containing hundreds to nearly one thousand species [11], [21]. When possible, future investigations into the phylogenetic dispersion of communities using phylogenies containing polytomies should generate a distribution of possible results by randomly resolving the polytomies in the phylogeny [6].

The last portion of this study analyzed whether the species richness of an assemblage compared to the number of terminal taxa in the phylogeny influenced the degree to which phylogenetic dispersion results were biased. There were no clear and consistent trends stemming from these analyses. The main result of interest came from the analyses using the largest phylogeny, where the NRI and NTI metrics were biased in opposing directions as the number of taxa in the assemblage increased. Specifically, power increased as the number of taxa increased when using NRI and the power decreased for NTI. This result is likely due to the NRI being calculated from pair-wise distances and NTI being calculated from nearest neighbor distances that are expected to be more sensitive to the degree of phylogenetic resolution (Fig. 1). Thus, increasing the number of taxa in the assemblage may stabilize the NRI metric and destabilize the NTI as the phylogeny becomes unresolved.

As the interest in including phylogenetic information into studies of species diversity and co-existence has outpaced our ability to generate fully resolved phylogenetic hypotheses for every study system, more and more researchers have begun to use phylogenies in their research that contain multiple unresolved nodes. It is clear that the use of phylogenies with multiple unresolved nodes is not the most desirable scenario, but it is likely to persist. Thus, it is now critical to quantify how this lack of resolution influences the metrics of phylogenetic diversity and dispersion and in what instances do we compromise the greatest amount of statistical power. The present analyses provide a first step towards explicitly quantifying these biases. In particular, I have shown that both phylogenetic diversity and dispersion metrics can be very sensitive to phylogenetic resolution when the phylogeny is large and when the lack of resolution is basal. Encouragingly, when the lack of resolution is terminal the loss of statistical power is greatly minimized. Lastly, the analyses indicate that researchers utilizing the metrics analyzed here are generally prone to underestimate the phylogenetic diversity and dispersion in communities when phylogenies are not completely resolved.

## Supporting Information

Table S1 (0.06 MB DOC)

Table S2 (0.07 MB DOC)

Table S3 (0.07 MB DOC)

Table S4 (0.07 MB DOC)

Table S5 (0.07 MB DOC)

Table S6 (0.07 MB DOC)

Figure S1 (0.46 MB DOC)

Figure S2 (0.45 MB DOC)

Figure S3 (0.09 MB DOC)

Figure S4 (0.18 MB DOC)
