# Supplementary material for: Phylogenetic Resolution and Quantifying the Phylogenetic Diversity and Dispersion of Communities
Source: PLoS One. 2009 Feb 5;4(2):e4390. doi: 10.1371/journal.pone.0004390 (PMC2633039; doi:10.1371/journal.pone.0004390)
Supplement: Table S1 — (0.06 MB DOC) [file pone.0004390.s001.doc]

**Table S1.** A table representing the power to predict MPD, MNND or FI of an assemblage with the maximal possible phylogenetic diversity estimated using the Greedy Algorithm. The slopes and r^2^ values from regressing the MPD, MNND or FI values derived using a randomly ‘unresolved’ phylogeny onto the MPD, MNND or FI values derived using a fully resolved phylogeny. The size of the phylogeny is represented by *N* and the percentage of nodes that were ‘unresolved’ is indicated by R_x_. Slopes less than one show a bias towards under-predicting the phylogenetic diversity in an assemblage and vice versa for slopes greater than one.

|  |  | **R_20_** | | **R_25_** | | **R_30_** | | **R_35_** | |
| --- | --- | --- | --- | --- | --- | --- | --- | --- | --- |
|  | N | ***m*** | ***r^2^*** | ***m*** | ***r^2^*** | ***m*** | ***r^2^*** | ***m*** | ***r^2^*** |
| MPD | 20 | 0.991 | 0.994 | 0.985 | 0.993 | 0.988 | 0.990 | 0.983 | 0.983 |
|  | 40 | 0.982 | 0.998 | 1.007 | 0.999 | 1.005 | 0.999 | 1.010 | 0.998 |
|  | 80 | 0.992 | 0.999 | 0.998 | 0.999 | 0.994 | 0.999 | 0.993 | 0.999 |
|  | 160 | 0.994 | 0.989 | 0.992 | 0.988 | 0.988 | 0.988 | 0.977 | 0.985 |
|  | 320 | 1.000 | 0.995 | 0.999 | 0.997 | 0.999 | 0.996 | 0.998 | 0.995 |
| **MNND** | 20 | 1.010 | 0.999 | 0.998 | 0.995 | 0.995 | 0.995 | 0.993 | 0.988 |
|  | 40 | 1.011 | 0.997 | 1.001 | 0.995 | 1.012 | 0.992 | 1.045 | 0.989 |
|  | 80 | 0.992 | 0.996 | 0.999 | 0.993 | 0.999 | 0.989 | 1.001 | 0.985 |
|  | 160 | 0.981 | 0.988 | 1.001 | 0.988 | 0.999 | 0.982 | 0.997 | 0.973 |
|  | 320 | 1.012 | 0.988 | 1.008 | 0.989 | 1.039 | 0.989 | 1.067 | 0.982 |
| **FI** | 20 | 0.939 | 0.999 | 0.933 | 0.999 | 0.870 | 0.998 | 0.869 | 0.998 |
|  | 40 | 0.923 | 0.995 | 0.920 | 0.994 | 0.877 | 0.994 | 0.833 | 0.994 |
|  | 80 | 0.923 | 0.996 | 0.909 | 0.996 | 0.888 | 0.995 | 0.836 | 0.995 |
|  | 160 | 0.911 | 0.995 | 0.873 | 0.995 | 0.868 | 0.994 | 0.864 | 0.993 |
|  | 320 | 0.910 | 0.997 | 0.863 | 0.992 | 0.855 | 0.989 | 0.852 | 0.990 |
