# Supplementary material for: Phylogenetic Resolution and Quantifying the Phylogenetic Diversity and Dispersion of Communities
Source: PLoS One. 2009 Feb 5;4(2):e4390. doi: 10.1371/journal.pone.0004390 (PMC2633039; doi:10.1371/journal.pone.0004390)
Supplement: Table S2 — (0.07 MB DOC) [file pone.0004390.s002.doc]

**Table S2.** A table representing the power to predict MPD, MNND or FI of an assemblage with the minimal possible phylogenetic diversity estimated using the Greedy Algorithm. The slopes and r^2^ values from regressing the MPD, MNND or FI values derived using a randomly ‘unresolved’ phylogeny onto the MPD, MNND or FI values derived using a fully resolved phylogeny. The size of the phylogeny is represented by *N* and the percentage of nodes that were ‘unresolved’ is indicated by R_x_. Slopes less than one show a bias towards under-predicting the phylogenetic diversity in an assemblage and vice versa for slopes greater than one.

|  |  | **R_20_** | | **R_25_** | | **R_30_** | | **R_35_** | |
| --- | --- | --- | --- | --- | --- | --- | --- | --- | --- |
|  | N | ***m*** | ***r^2^*** | ***m*** | ***r^2^*** | ***m*** | ***r^2^*** | ***m*** | ***r^2^*** |
| MPD | 20 | 0.993 | 0.995 | 0.989 | 0.995 | 0.988 | 0.992 | 0.985 | 0.989 |
|  | 40 | 0.990 | 0.996 | 0.990 | 0.997 | 1.001 | 0.996 | 0.999 | 0.991 |
|  | 80 | 0.993 | 0.998 | 0.991 | 0.998 | 0.993 | 0.996 | 0.990 | 0.988 |
|  | 160 | 0.994 | 0.998 | 0.999 | 0.996 | 0.996 | 0.994 | 0.986 | 0.985 |
|  | 320 | 0.993 | 0.994 | 0.991 | 0.994 | 0.995 | 0.991 | 0.996 | 0.984 |
| **MNND** | 20 | 1.009 | 0.998 | 1.008 | 0.998 | 1.010 | 0.997 | 1.013 | 0.992 |
|  | 40 | 1.011 | 0.999 | 1.011 | 0.998 | 1.013 | 0.998 | 1.037 | 0.985 |
|  | 80 | 1.011 | 0.995 | 1.013 | 0.996 | 1.009 | 0.990 | 1.052 | 0.983 |
|  | 160 | 1.017 | 0.991 | 1.023 | 0.990 | 1.039 | 0.983 | 1.052 | 0.977 |
|  | 320 | 1.023 | 0.992 | 1.040 | 0.988 | 1.035 | 0.980 | 1.077 | 0.979 |
| **FI** | 20 | 0.953 | 0.999 | 0.950 | 0.998 | 0.920 | 0.998 | 0.901 | 0.997 |
|  | 40 | 0.942 | 0.998 | 0.910 | 0.997 | 0.907 | 0.995 | 0.873 | 0.996 |
|  | 80 | 0.933 | 0.996 | 0.923 | 0.995 | 0.900 | 0.995 | 0.870 | 0.993 |
|  | 160 | 0.933 | 0.997 | 0.904 | 0.996 | 0.876 | 0.994 | 0.869 | 0.994 |
|  | 320 | 0.923 | 0.995 | 0.898 | 0.995 | 0.845 | 0.992 | 0.832 | 0.991 |
