# Supplementary material for: Phylogenetic Resolution and Quantifying the Phylogenetic Diversity and Dispersion of Communities
Source: PLoS One. 2009 Feb 5;4(2):e4390. doi: 10.1371/journal.pone.0004390 (PMC2633039; doi:10.1371/journal.pone.0004390)
Supplement: Table S3 — (0.07 MB DOC) [file pone.0004390.s003.doc]

**Table S3.** A table representing the power to predict MPD, MNND or FI of randomly generated assemblages. The slopes and r^2^ values from regressing the MPD, MNND or FI values derived using a randomly ‘unresolved’ phylogeny onto the MPD, MNND or FI values derived using a fully resolved phylogeny. The size of the phylogeny is represented by *N* and the percentage of nodes that were ‘unresolved’ is indicated by R_x_. Slopes less than one show a bias towards under-predicting the phylogenetic diversity in an assemblage and vice versa for slopes greater than one.

|  |  | **R_20_** | | **R_25_** | | **R_30_** | | **R_35_** | |
| --- | --- | --- | --- | --- | --- | --- | --- | --- | --- |
|  | N | ***m*** | ***r^2^*** | ***m*** | ***r^2^*** | ***m*** | ***r^2^*** | ***m*** | ***r^2^*** |
| MPD | 20 | 1.016 | 0.994 | 1.010 | 0.993 | 1.007 | 0.993 | 0.997 | 0.989 |
|  | 40 | 1.007 | 0.999 | 1.007 | 0.999 | 1.005 | 0.999 | 1.010 | 0.999 |
|  | 80 | 0.996 | 1.000 | 0.998 | 1.000 | 0.994 | 1.000 | 0.993 | 1.000 |
|  | 160 | 0.950 | 0.975 | 1.006 | 0.999 | 0.989 | 0.999 | 0.964 | 0.994 |
|  | 320 | 1.003 | 0.995 | 1.002 | 0.995 | 1.002 | 0.995 | 1.002 | 0.995 |
| **MNND** | 20 | 1.015 | 0.995 | 0.998 | 0.992 | 0.995 | 0.990 | 0.993 | 0.983 |
|  | 40 | 1.019 | 0.996 | 1.022 | 0.995 | 1.020 | 0.994 | 1.045 | 0.991 |
|  | 80 | 0.990 | 0.992 | 1.008 | 0.991 | 1.001 | 0.989 | 1.003 | 0.988 |
|  | 160 | 0.973 | 0.978 | 1.033 | 0.997 | 0.984 | 0.984 | 0.952 | 0.961 |
|  | 320 | 1.026 | 0.985 | 1.035 | 0.986 | 1.047 | 0.985 | 1.052 | 0.985 |
| **FI** | 20 | 0.944 | 0.998 | 0.923 | 0.996 | 0.892 | 0.996 | 0.862 | 0.992 |
|  | 40 | 0.916 | 0.994 | 0.882 | 0.993 | 0.857 | 0.992 | 0.829 | 0.990 |
|  | 80 | 0.920 | 0.998 | 0.890 | 0.997 | 0.871 | 0.998 | 0.853 | 0.997 |
|  | 160 | 0.917 | 0.998 | 0.888 | 0.997 | 0.863 | 0.997 | 0.841 | 0.997 |
|  | 320 | 0.906 | 0.996 | 0.876 | 0.993 | 0.850 | 0.994 | 0.827 | 0.995 |
