# Supplementary material for: Phylogenetic Resolution and Quantifying the Phylogenetic Diversity and Dispersion of Communities
Source: PLoS One. 2009 Feb 5;4(2):e4390. doi: 10.1371/journal.pone.0004390 (PMC2633039; doi:10.1371/journal.pone.0004390)
Supplement: Table S4 — (0.07 MB DOC) [file pone.0004390.s004.doc]

**Table S4.** A table representing the power to predict MPD, MNND or FI of an assemblage with the maximal possible phylogenetic diversity estimated using the Greedy Algorithm. The slopes and r^2^ values from regressing the MPD, MNND or FI values derived using a terminally ‘unresolved’ phylogeny onto the MPD, MNND or FI values derived using a fully resolved phylogeny. The size of the phylogeny is represented by *N* and the percentage of nodes that were ‘unresolved’ is indicated by R_x_. Slopes less than one show a bias towards under-predicting the phylogenetic diversity in an assemblage and vice versa for slopes greater than one.

|  |  | **T_20_** | | **T_25_** | | **T_30_** | | **T_35_** | |
| --- | --- | --- | --- | --- | --- | --- | --- | --- | --- |
|  | N | ***m*** | ***r^2^*** | ***m*** | ***r^2^*** | ***m*** | ***r^2^*** | ***m*** | ***r^2^*** |
| MPD | 20 | 0.999 | 0.999 | 0.999 | 0.998 | 0.998 | 0.998 | 0.995 | 0.996 |
|  | 40 | 0.999 | 0.999 | 0.998 | 0.999 | 0.999 | 0.998 | 1.000 | 0.998 |
|  | 80 | 1.000 | 0.999 | 0.999 | 0.998 | 1.000 | 0.998 | 1.000 | 0.995 |
|  | 160 | 0.998 | 0.999 | 0.997 | 0.999 | 0.998 | 0.999 | 0.998 | 0.999 |
|  | 320 | 0.999 | 0.998 | 0.999 | 0.999 | 0.999 | 0.999 | 1.000 | 0.997 |
| **MNND** | 20 | 1.000 | 0.999 | 1.001 | 0.999 | 1.002 | 0.999 | 1.002 | 0.998 |
|  | 40 | 1.000 | 0.999 | 1.000 | 0.999 | 1.002 | 0.998 | 1.003 | 0.999 |
|  | 80 | 1.000 | 0.999 | 1.000 | 0.999 | 1.001 | 0.998 | 1.006 | 0.998 |
|  | 160 | 1.003 | 0.999 | 1.002 | 0.998 | 1.003 | 0.998 | 1.006 | 0.998 |
|  | 320 | 1.004 | 0.999 | 1.004 | 0.998 | 1.004 | 0.997 | 1.006 | 0.996 |
| **FI** | 20 | 0.999 | 0.999 | 0.997 | 0.999 | 0.988 | 0.999 | 0.981 | 0.998 |
|  | 40 | 0.998 | 0.999 | 0.988 | 0.999 | 0.984 | 0.999 | 0.974 | 0.998 |
|  | 80 | 0.996 | 0.999 | 0.989 | 0.999 | 0.985 | 0.998 | 0.977 | 0.996 |
|  | 160 | 0.986 | 0.999 | 0.979 | 0.998 | 0.975 | 0.997 | 0.969 | 0.996 |
|  | 320 | 0.987 | 0.999 | 0.979 | 0.998 | 0.971 | 0.997 | 0.968 | 0.998 |
