# Supplementary material for: Phylogenetic Resolution and Quantifying the Phylogenetic Diversity and Dispersion of Communities
Source: PLoS One. 2009 Feb 5;4(2):e4390. doi: 10.1371/journal.pone.0004390 (PMC2633039; doi:10.1371/journal.pone.0004390)
Supplement: Table S6 — (0.07 MB DOC) [file pone.0004390.s006.doc]

**Table S6.** A table representing the power to predict MPD, MNND or FI of randomly generated assemblages. The slopes and r^2^ values from regressing the MPD, MNND or FI values derived using a terminally ‘unresolved’ phylogeny onto the MPD, MNND or FI values derived using a fully resolved phylogeny. The size of the phylogeny is represented by *N* and the percentage of nodes that were ‘unresolved’ is indicated by R_x_. Slopes less than one show a bias towards under-predicting the phylogenetic diversity in an assemblage and vice versa for slopes greater than one.

|  |  | **T_20_** | | **T_25_** | | **T_30_** | | **T_35_** | |
| --- | --- | --- | --- | --- | --- | --- | --- | --- | --- |
|  | N | ***m*** | ***r^2^*** | ***m*** | ***r^2^*** | ***m*** | ***r^2^*** | ***m*** | ***r^2^*** |
| MPD | 20 | 1.000 | 1.000 | 0.999 | 0.999 | 0.994 | 0.999 | 0.987 | 0.997 |
|  | 40 | 1.000 | 1.000 | 0.999 | 1.000 | 1.000 | 1.000 | 0.999 | 1.000 |
|  | 80 | 1.000 | 1.000 | 1.001 | 1.000 | 1.000 | 1.000 | 1.001 | 1.000 |
|  | 160 | 1.000 | 1.000 | 1.000 | 1.000 | 1.000 | 1.000 | 1.001 | 1.000 |
|  | 320 | 1.007 | 0.996 | 1.007 | 0.996 | 1.007 | 0.996 | 1.007 | 0.996 |
| **MNND** | 20 | 1.001 | 0.999 | 1.000 | 0.998 | 0.989 | 0.996 | 0.977 | 0.990 |
|  | 40 | 1.003 | 1.000 | 1.004 | 0.997 | 1.005 | 0.996 | 1.006 | 0.996 |
|  | 80 | 1.006 | 0.999 | 1.010 | 0.999 | 1.017 | 0.998 | 1.021 | 0.997 |
|  | 160 | 1.006 | 1.000 | 1.011 | 1.000 | 1.017 | 0.999 | 1.031 | 0.998 |
|  | 320 | 1.019 | 0.993 | 1.027 | 0.993 | 1.031 | 0.993 | 1.042 | 0.992 |
| **FI** | 20 | 0.965 | 1.000 | 0.945 | 0.998 | 0.919 | 0.997 | 0.906 | 0.996 |
|  | 40 | 0.974 | 1.000 | 0.949 | 0.999 | 0.929 | 0.998 | 0.908 | 0.998 |
|  | 80 | 0.971 | 1.000 | 0.954 | 0.999 | 0.931 | 0.999 | 0.907 | 0.999 |
|  | 160 | 0.977 | 1.000 | 0.960 | 1.000 | 0.939 | 0.999 | 0.906 | 0.999 |
|  | 320 | 0.963 | 1.000 | 0.945 | 0.999 | 0.929 | 0.998 | 0.906 | 0.998 |
