# Supplementary material for: Phylogenetic Resolution and Quantifying the Phylogenetic Diversity and Dispersion of Communities
Source: PLoS One. 2009 Feb 5;4(2):e4390. doi: 10.1371/journal.pone.0004390 (PMC2633039; doi:10.1371/journal.pone.0004390)

**Figure S1.** A figure showing the power to predict NRI and NTI of an assemblage with the maximal possible phylogenetic diversity estimated using the Greedy Algorithm. The slopes and r^2^ values from regressing the NRI and NTI values derived using a terminally‘unresolved’ phylogeny onto the NRI and NTI values derived using a fully resolved phylogeny. The size of the phylogeny is represented by color and dashing of the lines. Specifically, the number of terminal taxa was 20 (finely dashed grey line), 40 (thickly dashed grey line), 80 (solid grey line), 160 (dashed black line), and 320 (solid black line). The percentage of nodes that were ‘unresolved’ is indicated by T_x_ on the x-axis. Slopes less than one show a bias towards under-predicting the phylogenetic diversity in an assemblage and vice versa for slopes greater than one (see Figure 1).


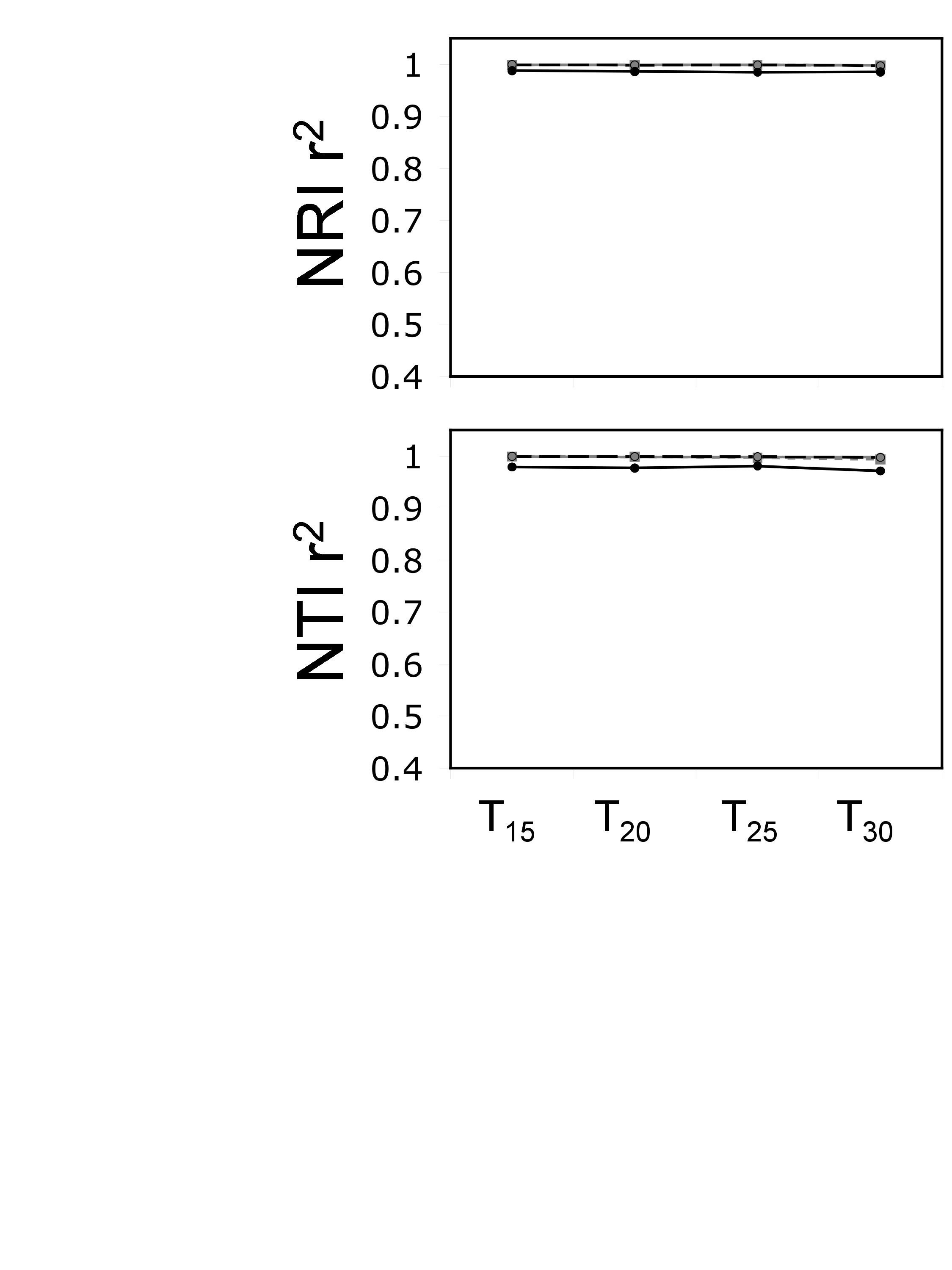

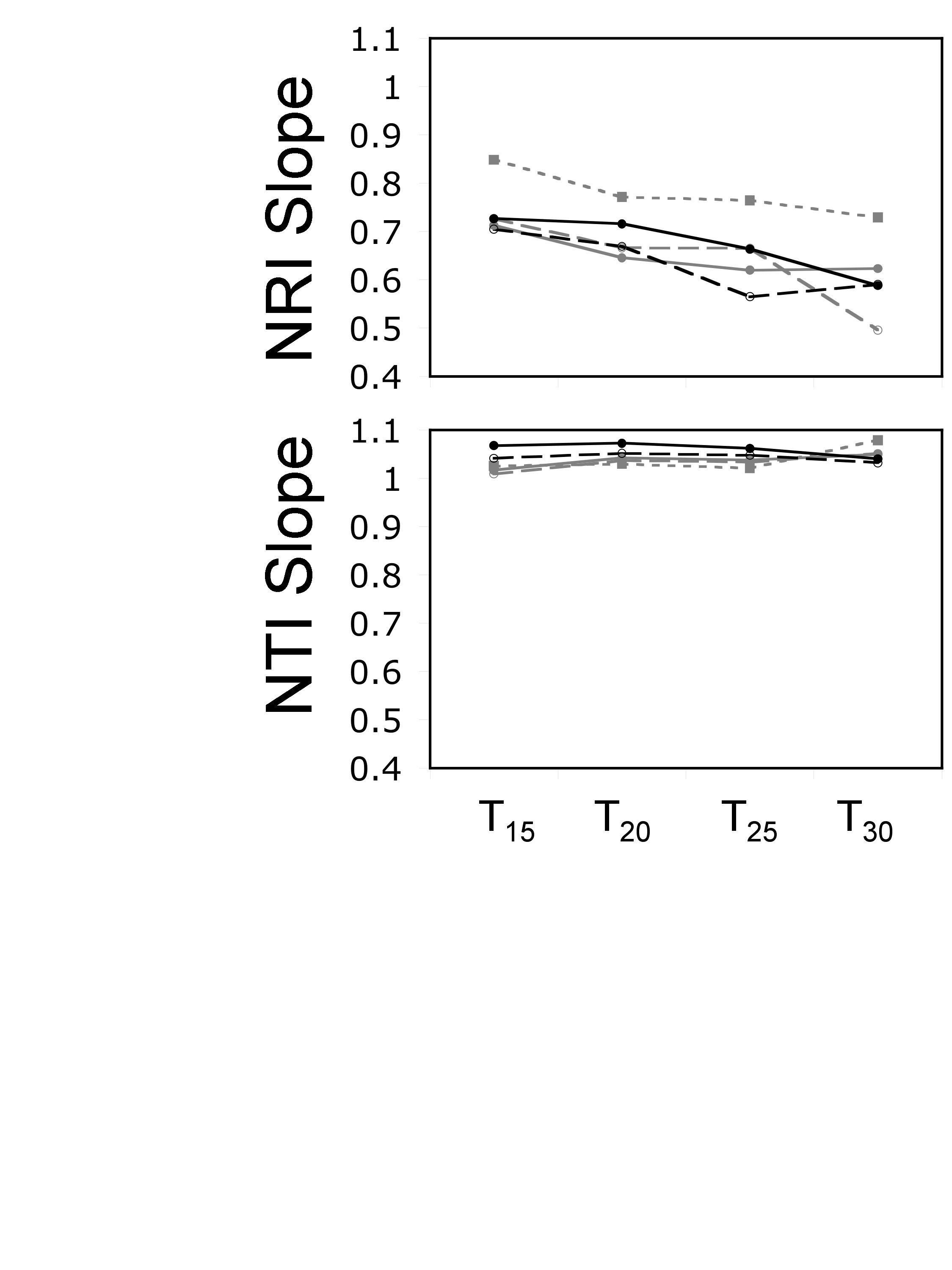

Supplement: Figure S1 — (0.46 MB DOC) [file pone.0004390.s007.doc]
