# Supplementary material for: Phylogenetic Resolution and Quantifying the Phylogenetic Diversity and Dispersion of Communities
Source: PLoS One. 2009 Feb 5;4(2):e4390. doi: 10.1371/journal.pone.0004390 (PMC2633039; doi:10.1371/journal.pone.0004390)

**Figure S4.** Box-plots showing the power to predict NRI and NTI of randomly generated assemblages with different species diversities (x-axis). The slopes and r^2^ values from regressing the NRI and NTI values derived using a randomly (top two rows) or a terminally (bottom two rows) ‘unresolved’ phylogeny onto the NRI and NTI values derived using a fully resolved phylogeny. The size of the phylogeny (N_TIPS_) varies in the columns from the left to right. Slopes less than one show a bias towards under-predicting the phylogenetic diversity in an assemblage and vice versa for slopes greater than one (see Figure 1).


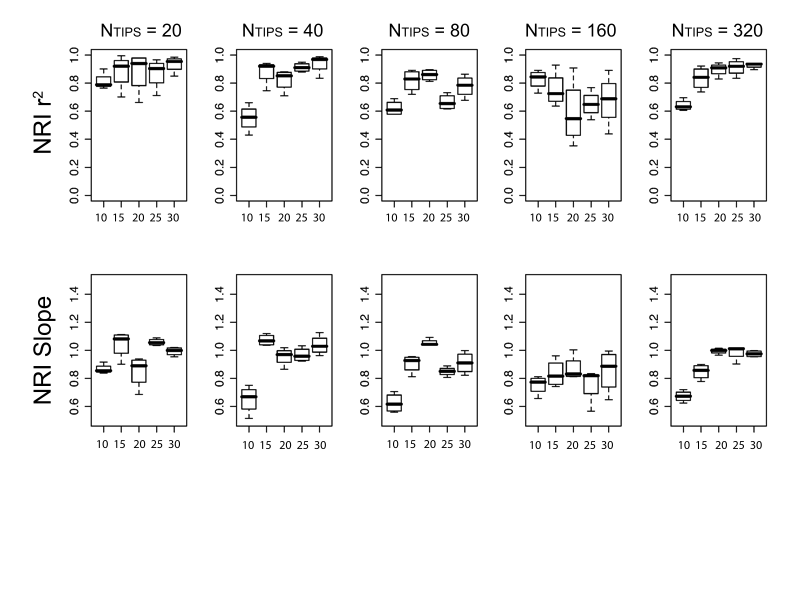

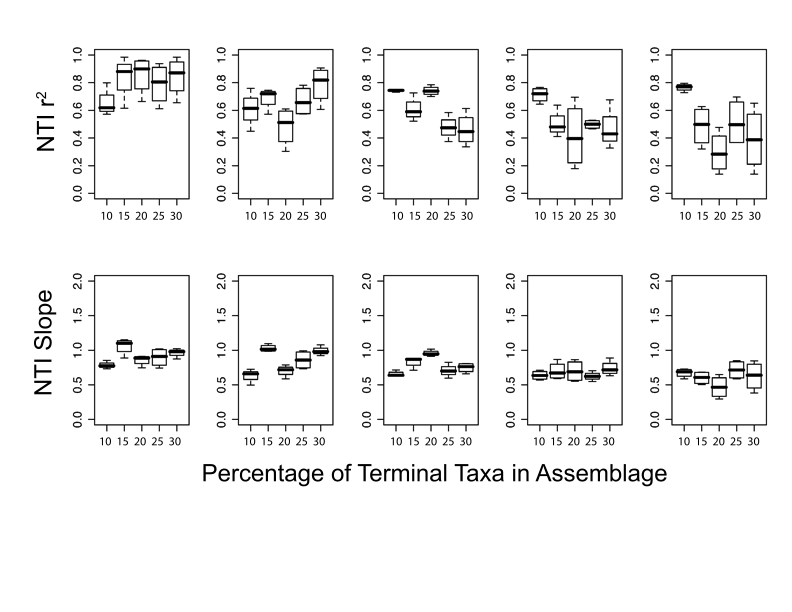

Supplement: Figure S4 — (0.18 MB DOC) [file pone.0004390.s010.doc]
